# Tyrosinase-catalyzed site-specific immobilization of engineered C-phycocyanin to surface

**DOI:** 10.1038/srep05370

**Published:** 2014-06-20

**Authors:** Greta Faccio, Michael M. Kämpf, Chiara Piatti, Linda Thöny-Meyer, Michael Richter

**Affiliations:** 1 Empa, Swiss Federal Laboratories for Materials Science and Technology - Laboratory for Bioactive Materials, Lerchenfeldstrasse 5, 9014 St. Gallen, Switzerland; 2 Dipartimento di Biotecnologie e Scienze della Vita, Università degli Studi dell'Insubria, via J.H. Dunant 3, 21100 Varese, Italy

## Abstract

Enzymatic crosslinking of proteins is often limited by the steric availability of the target residues, as of tyrosyl side chains in the case of tyrosinase. Carrying an N-terminal peptide-tag containing two tyrosine residues, the fluorescent protein C-phycocyanin HisCPC from *Synechocystis* sp. PCC6803 was crosslinked to fluorescent high-molecular weight forms with tyrosinase. Crosslinking with tyrosinase in the presence of L-tyrosine produced non fluorescent high-molecular weight products. Incubated in the presence of tyrosinase, HisCPC could also be immobilized to amino-modified polystyrene beads thus conferring a blue fluorescence. Crosslinking and immobilization were site-specific as both processes required the presence of the N-terminal peptide in HisCPC.

Protein crosslinking can be achieved with enzymes that catalyse the formation of new covalent bonds between substrate proteins^1^. For example, bacterial and fungal tyrosinases can be used to crosslink various substrate proteins in isolated form, e.g. milk caseins, and within a complex matrix, e.g. in milk and bread systems^2^,^3^,^4^. Tyrosinase catalyzes the *ortho*-hydroxylation of monophenolic compounds such as tyrosyl groups of proteins and the further oxidation to *ortho*-quinone species. Once tyrosinase activates tyrosines to *ortho*-quinones, these can further react non-enzymatically with lysyl, tyrosyl, cysteinyl or histidyl side chain within the same or a different polypeptide chain, thus forming intra- or inter-molecular covalent bonds, respectively^1^. Tyrosinase has preferred crosslinking activity on proteins with a loose three-dimensional structure, whereas stably folded proteins are usually not good substrates. Studies report the overcoming of this limitation by carrying out the crosslinking reaction in the presence of a small phenolic compound such as phenol, or caffeic acid^5^ that can act as crosslinking-promoting agent. This approach made the production of crosslinked forms of industrially interesting enzymes, also known as crosslinked enzyme aggregates (CLEAs), possible. For example, we reported production of crosslinked aggregates of *Candida antarctica* lipase (CALB) with tyrosinase from *Verrucomicrobium spinosum* in the presence of phenol^5^.

However, few studies have addressed the possibility of directing the crosslinking reaction towards materials, thus aiming at the functionalization of the surface with proteins.

Light absorbing and fluorescent phycobiliproteins such as the photosynthethic blue fluorescent C-phycocyanin from Cyanobacteria have attracted increasing attention and are subject of many patent applications^6^. This is not only due to their light-harvesting ability that has been exploited in electrochemical devices such as photodetectors and photovoltaic cells^7^,^8^, but also because of their antioxidant^9^, antidiabetic^10^, and antitumoral^11^,^12^,^13^,^14^ properties. Among the latest technological applications, C-phycocyanins have been used to functionalise photoelectrochemical cells for hydrogen production^8^ and have been immobilized on chitosomes for pharmaceutical purposes^15^. Phycobiliproteins are water-soluble and have been used as fluorescent probes for diagnostic tools, e.g. labelled antibodies in immunoassays. Moreover, they can be used to label antibodies for cell-sorting by flow cytometry, and fluorescence detection after *in situ* hybridization by fluorescence microscopy. For example, allophycocyanin (104 kDa) from *Spirulina* sp. is sold in a more stable, crosslinked form with a commercial value of 70–1400 USD/mg of protein. Crosslinked allophycocyanin is produced by using chemical crosslinkers such as glutaraldehyde or formaldehyde that need to be removed before use. Similarly, chemical crosslinking with formaldehyde or DSP [dithiobis(succinimidyl propionate)] has been used to produce aggregates of C-phycocyanins with higher thermal stability^16^,^17^.

Firstly, the aim of this work was the characterisation of recombinantly expressed α-subunit of C-phycocyanin from *Synechocystis* sp. PCC6803 (HisCPC), with respect to the commercial equivalent preparation from *Spirulina* sp. (CPC). Secondly, the site-specific immobilization of HisCPC in a crosslinked form and on a solid surface using the bacterial tyrosinase from *V. spinosum* was carried out.

## Results

### Overexpression and purification of HisCPC in *E. coli*

In order to obtain sufficient amounts of HisCPC for the crosslinking studies, the α-subunit of C-phycocyanin from *Synechocystis* sp. PCC6803 was produced in *E. coli* with the system developed by Glazer's group^18^ with an optimised procedure. The method was based on a two-plasmid system harbouring not only the gene coding for the apoprotein of the α subunit of C-phycocyanin but also four additional genes, i.e. *cpcF* and *cpcE* coding for a heterodimeric lyase responsible for cofactor attachment and *HO1* and *pcyA* coding for heme oxygenase 1 and 3Z-phycocyanobilin:ferredoxin oxidoreductase, respectively, for the covalent attachment of the tetrapyrrole cofactor phycobilin. In a first approach, three different media, namely standard (LB medium), rich and buffered (TB medium), and rich and non-buffered (SB medium) nutrient conditions were compared. 20 h after induction, colourless cells and a final OD_600_ of 1 were obtained from cultures grown in LB medium, whereas light blue cells and an OD_600_ of 1.5 were obtained from cultures cultivated in SB medium. A constant progression in blue colour formation was observed for cultivation in TB medium, which reached a final OD_600_ of 2.2. SDS PAGE analysis of cells at different time points showed that cultivation in TB medium lead to the highest accumulation of HisCPC visible as a ~20 kDa protein band (calculated molecular mass of 20.46 kDa) with respect to the total protein content (Supplementary Information S1 online). The concomitant accumulation of a ~25 kDa protein was observed and attributed to the expression of *cpcE* whose gene product has a calculated molecular mass of 23.2 kDa. Accumulation of HisCPC resulted in a strong fluorescence of all cells cultured for 20 h after induction in TB medium (Supplementary Information S1 online). Consequently, TB medium was chosen for the next step of optimization.

A shift to static condition after induction has been reported to promote cofactor attachment of various proteins^19^,^20^. We thus investigated the effect of static conditions on HisCPC production. At induction, cultures were transferred to static conditions and incubated at different temperatures for 20 h. The production level of HisCPC was determined in the cell free extracts and in the purified sample. Incubation at 22°C under static conditions post-induction was the optimal condition for production of HisCPC according to our test experiments and resulted in the highest production level (0.37 mg/ml) and an A_620_/A_280_ ratio of 1.3 of the purified sample (Supplementary Information S2 online).

### Biochemical properties of HisCPC

**Figure 1:**
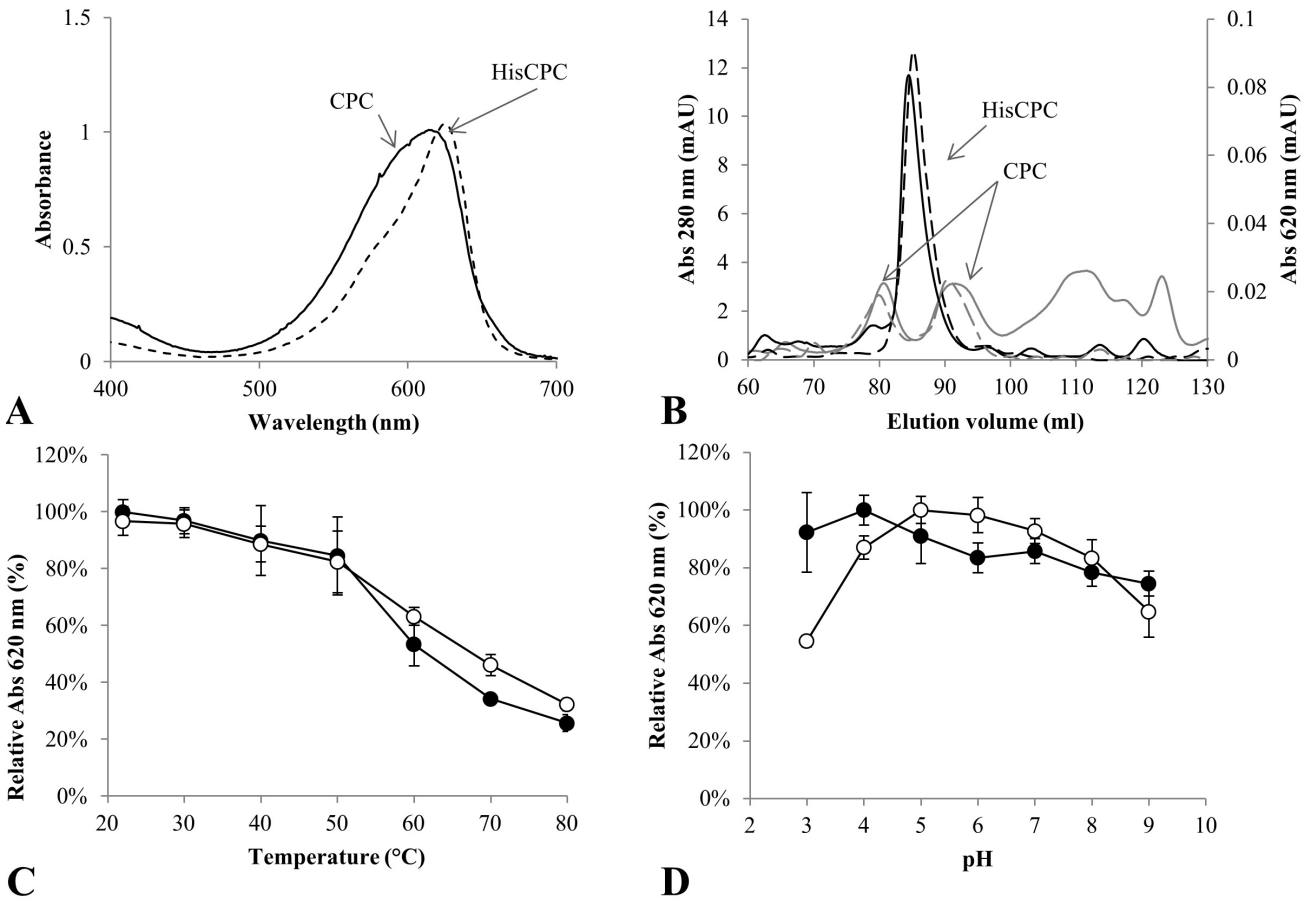
(A) UV-Vis absorption spectra of CPC from *Spirulina* sp. (continuous line) and the recombinant HisCPC from *Synechocystis* sp. PCC6803 (dotted line). Spectra are normalized to an absorbance of 1 at 620 nm. (B) Size-exclusion chromatography of CPC (grey lines) and HisCPC (black lines). Proteins were separated with a HiLoad 16/600 Superdex 200 prep grade column and the elution was monitored as absorbance at 280 nm (continuous line) and at 620 nm (dotted line). (C) Temperature and (D) pH profile of CPC (empty circles) and HisCPC (filled circles). Proteins were incubated under different conditions for 5 min. Values are reported as average ± standard deviation of three samples.

Purified HisCPC and the commercially available CPC from *Spirulina* sp. were compared on the basis of their UV-Vis absorption, multimeric state, and stability at different pH values and temperatures. Both proteins absorbed in the visible range at around 620 nm. CPC had a broad absorbance peak with a maximum at 615 nm, resulting from the overlay of the absorption of the α-subunit at 620 nm and the β-subunit at 608 nm^21^, whereas HisCPC, as constituted by the only α subunit, had a sharp absorbance peak with a maximum at 626 nm and a shoulder around 570 nm (Figure 1A).

Size-exclusion chromatography (SEC) was used to assess the multimeric state of both HisCPC and the commercial CPC in solution. C-phycocyanin α- and β-subunits are reported to associate into heterodimers and further into trimers of dimers. In contrast to HisCPC that eluted as a single peak at 83.7 ml corresponding to a molecular mass of 26.0 ± 3.8 kDa, CPC eluted in two peaks at 80.1 and 90.5 ml corresponding to molecular masses of 36.1 ± 2.7 and 13.8 ± 1.0 kDa, possibly representing an α/β-dimer and a single α or β subunit, respectively (Figure 1B). The ratio between the areas of the phycocyanin-containing peaks and the total area of the eluted sample indicated a higher purity of the HisCPC preparation: this protein constituted 82 ± 2% of the total protein as opposed to the CPC sample that constituted only 46 ± 6% of the total protein.

In order to evaluate the stability features of HisCPC and CPC, these were incubated under different temperature and pH conditions. Both proteins retained more than 80% of the initial absorbance at 620 nm when incubated for 5 min at temperatures up to 50°C, whereas at higher temperatures CPC showed a slightly increased thermal stability. Accordingly, a slightly higher residual fluorescence was detected for CPC that retained 53, 37 and 21% of the initial fluorescence compared to the 32, 5, and 3% retained by HisCPC when incubated at 60, 70, and 80°C, respectively. HisCPC was less sensitive to pH than CPC and retained its characteristic absorbance and fluorescence at acidic conditions, e.g. at pH 3 and 4 (Figure 1D, Supplementary Information S3 online).

### Crosslinking of HisCPC with tyrosinase

**Figure 2:**
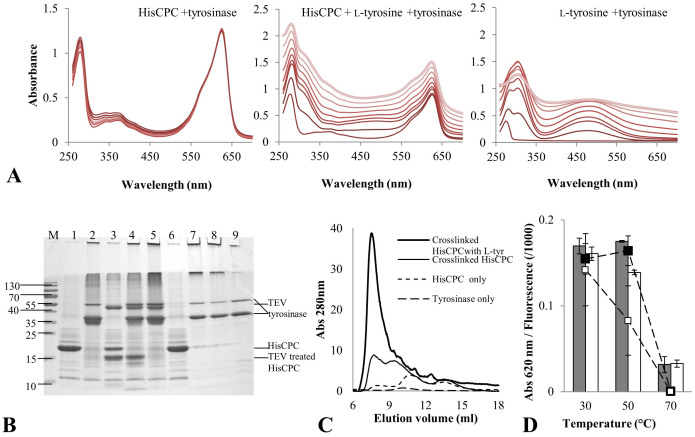
Tyrosinase catalyzed crosslinking of HisCPC. (A) UV-Vis spectra monitoring the direct crosslinking of HisCPC (left panel) and crosslinking in the presence of L-tyrosine (middle panel) with tyrosinase VsTYR. For comparison, the oxidation of L-tyrosine with tyrosinase is also reported (right panel). Reactions were carried out in 100 mM K-phosphate pH 6.8 buffer. The periodic measurement of the absorbance spectrum is shown by shading from dark (initial conditions) to light grey (final conditions after 70 min). (B) SDS PAGE of HisCPC upon crosslinking in the presence or absence of L-tyrosine with tyrosinase. HisCPC (lane 1, 5 μg) was incubated in the presence of tyrosinase (lane 2), subjected to partial digestion with TEV (lane 3) and subsequently incubated in the presence of tyrosinase (lane 4), crosslinked with tyrosinase and subsequently incubated with TEV (lane 5), incubated with L-tyrosine (lane 6), and of both L-tyrosine and tyrosinase (lane 7). The product of incubation of L-tyrosine and tyrosinase (lane 8) and tyrosinase only (lane 9) are also shown). Molecular weight markers (lane M) are in kDa. (C) Size-exclusion chromatography of HisCPC in native and crosslinked form in the presence or absence of L-tyrosine. Separation was performed using a 17 ml volume column packed with Superdex 75 resin and a 1 ml/min flow of 100 mM potassium phosphate pH 7.5, 150 mM NaCl at 22°C. (D) Thermal stability of directly crosslinked HisCPC. Absorbance values at 620 nm (bars) and fluorescence values (square markers) were measured after 15 min incubation at different temperatures for native (filled bar and marker) and crosslinked (empty bar and marker) HisCPC.

Crosslinked forms of fluorescent proteins such as C-phycocyanin are generally prepared with chemical crosslinkers and used in various applications as fluorescent labels. We investigated the applicability of the crosslinking enzyme tyrosinase to produce high-molecular weight forms of HisCPC. As stably folded and conformationally restricted proteins are generally poor substrates for tyrosinase, we first evaluated the use of auxiliary phenolic compounds. Crosslinking was performed by incubating the substrate protein HisCPC with tyrosinase in the presence or absence of 0.55 mM L-tyrosine in 100 mM K-phosphate at pH 6.8 for 1 h. The crosslinking reactions and the final products were analysed by UV-Vis spectroscopy, fluorescence-spectroscopy, size-exclusion chromatography, and SDS PAGE. The presence of L-tyrosine during crosslinking of HisCPC with tyrosinase led to an increase of absorbance at all wavelengths, due to the formation of dark-coloured high molecular mass precipitates that could be easily recovered by centrifugation leaving a colourless protein-free supernatant. The formation of the melanin precursor dopachrome was detected by its characteristic absorption peak at 475 nm^23^ (Figure 2A). On the contrary, incubation of HisCPC with tyrosinase alone led to an increase in absorbance at 310–320 nm (Figure 2A). The fluorescence of HisCPC subjected to direct crosslinking without additional tyrosine was retained. By contrast, significant loss of fluorescence was measured for crosslinked CPC in samples containing also 0.55 mM L-tyrosine, e.g. 110 ± 30% *versus* 13 ± 8% of the initial fluorescence after 70 min. Similar results were also obtained at lower protein concentrations, e.g. 0.2 mg/ml *versus* 0.5 mg/ml, for CPC, and crosslinking with L-tyrosine and tyrosinase led to a 5-fold faster loss of fluorescence (Supplementary Information S4 online).

SDS PAGE and SEC analyses of the reaction mixtures (Figure 2B and C, respectively) showed the complete crosslinking of HisCPC by tyrosinase, which resulted in the formation of covalent dimers and high molecular weight protein aggregates (Figure 2B, lane 2). This direct crosslinking preserved the blue colour of the protein solution and produced crosslinked products that migrated in a predominant band at ~40 kDa (HisCPC dimer) and a higher molecular weight smear. Under non-denaturing conditions in SEC, direct crosslinking of HisCPC lead to the formation of two major species with masses of ~282 kDa, eluting at 7.7 ml, and ~80 kDa, eluting at 9.4 ml, respectively (Figure 2C). When the small phenolic compound L-tyrosine was added to the reaction, the shift of the protein bands to higher molecular weights increased (Figure 2B, lane 7), and after SEC a major soluble species eluting at 7.5 ml with a calculated mass of ~320 kDa (Figure 2C) was obtained. The band at the interface between stacking and separating gel was visible in the unstained gel and showed that some aggregates could not enter the separating gel. Moreover, visible bands at the front of the stacking gel are likely due to the melanin formed from L-tyrosine in the presence of tyrosinase (Figure 2B, lanes 7 and 8). Protein bands corresponding to tyrosinase were visible in all crosslinked samples suggesting that the enzyme is not integrated into the crosslinked aggregates of HisCPC during the reaction, even in the presence of tyrosine (Figure 2B, lane 2,4,5,7,8). Direct crosslinking did not significantly affect the temperature stability of HisCPC compared to the one of the native form when incubation was carried out at 30°C for 15 min. Incubation at 70°C under analogous conditions caused precipitation of the protein as a colourless particulate abolishing both absorbance and fluorescence (Figure 2D).

The 186 amino acid-long HisCPC included a 24 amino acid N-terminal peptide (MGH_6_DYDIPTTENLYFQGAH) containing two tyrosine residues in addition to a hexa-histidine tag and a TEV recognition site for its removal (GenBank ID: AAP43514.1). In fact, the second tyrosine forms part of the TEV-protease recognition site and it is removed after cleavage, as cleavage occurs between Q and G. We proceeded to determine whether crosslinking by tyrosinase involved tyrosine residues from the original CPC molecule or from the N-terminal peptide. Thus, treatment with TEV protease of HisCPC was carried out before and after crosslinking, and the samples were analysed by SDS PAGE. Partial digestion with TEV in order to remove the N-terminal peptide lead to the formation of a species with lower molecular mass around 15 kDa (Figure 2B, lane 3) that was not consumed upon incubation with tyrosinase (Figure 2B, lane 4). We concluded that crosslinking by tyrosinase proceeds through the activation of at least one of the tyrosines in the N-terminal peptide when crosslinking through exposed tyrosine residues of the backbone due to conformational changes caused by the tyrosine tag is excluded. Although HisCPC carries various exposed tyrosine resides in its core domain (Supplementary Information S5 online), e.g. Tyr90, 97 and 110, these were apparently not available to tyrosinase for crosslinking. Crosslinked aggregates were not dissolved by treatment with TEV protease (Figure 2, lane 5). We concluded that the TEV recognition sites were blocked and/or not sterically available to the protease when HisCPC was in the crosslinked form. Note that the cleaved N-terminal peptide was still present in the crosslinking mixture; however, even if it was activated by tyrosinase it did not further react with residues from the globular HisCPC moiety to promote the formation of high molecular weight aggregates, i.e. it did not play a role similar to that of L-tyrosine.

### immobilization of HisCPC with tyrosinase

**Figure 3:**
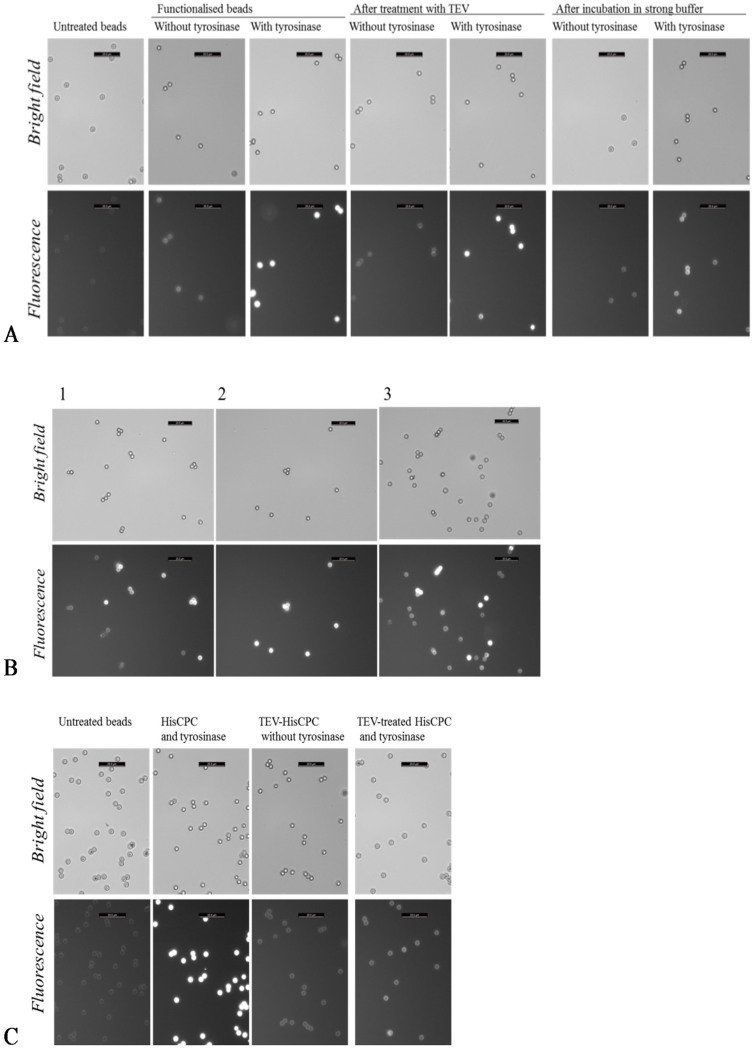
HisCPC immobilized on amino-modified beads using tyrosinase. (A) Beads were first incubated in the presence of a partially purified HisCPC preparation and with and without tyrosinase, and then treated with either TEV-protease or a strong buffer containing 100 mM NaCl and 0.1% v/v Tween 20 for 4 h. (B) Microscopy images of combinations of differently treated amino-modified polystyrene beads. Sample 1, mix of adsorbed beads plus washed crosslinked beads. Sample 2, mix of crosslinked beads with and without washing. Sample 3, mix of adsorbed beads and cross-linked, protease treated beads. (C) Beads were incubated with tyrosinase and HisCPC full-length or previously treated with TEV-protease. The scale bar on the top right corner of each figure corresponds to 20 μm. Images were taken with a fluorescence microscope under bright light (top rows) and with fluorescence using a N21 filter (λ_ex_ = 515–560 nm, λ_em_ ≥ 590 nm, bottom rows).

The availability of two tyrosine residues from the N-terminal peptide of HisCPC for crosslinking by tyrosinase suggested that, upon activation by tyrosinase, these could be directed towards surfaces to allow immobilization of the protein. Amino-modified polystyrene beads were incubated in the presence of HisCPC and tyrosinase. They acquired a distinct fluorescence, higher than what was recorded for samples incubated in absence of tyrosinase when only adsorption of HisCPC to the polymeric surfaces should occur (adsorbed beads). This indicated that HisCPC could be immobilized on the beads (crosslinked beads, Figure 3A). When these functionalized beads were washed with a buffer containing high salt concentration and a detergent (100 mM K-phosphate at pH 6.8 containing 100 mM NaCl, 0.1% v/v Tween 20), some but not all the fluorescence was lost (Figure 3B, sample 2). The retained fluorescence was still higher than what observed for beads with simply adsorbed HisCPC. This was confirmed in a mixture of adsorbed and crosslinked, washed beads, where two populations were clearly distinguishable (Figure 3B, sample 1). The loss of fluorescence could be explained by low stability of HisCPC in the washing buffer used, as HisCPC in this buffer retained 35% of the A_620_ retained in 100 mM K-phosphate pH 6.8 after incubation under analogous conditions. Treatment of crosslinked and adsorbed beads with TEV-protease did not significantly affect their fluorescence (Figure 3A) and, when combined, two types of beads with different fluorescence intensities were detected (Figure 3B, sample 3). Incubation of the beads with TEV-treated HisCPC and tyrosinase produced beads with a level of fluorescence visually more similar to adsorbed beads than crosslinked beads (Figure 3C).

**Figure 4:**
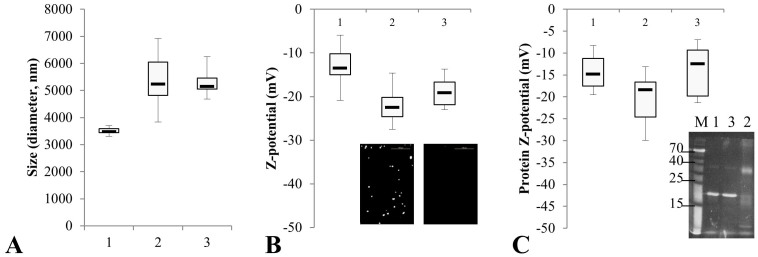
Box-and-whiskers plot showing the size (A) and Z-potential (B) of amino-modified beads untreated (sample 1), incubated with pure HisCPC and tyrosinase (sample 2), and incubated only with HisCPC (sample 3). In panel B, the inset shows the beads as imaged by fluorescence microscopy. Z-potential measurement (C) and SDS PAGE analysis (inset, staining with zinc) of the protein solution at the end of the immobilization process (samples numbered as in A and B).

Beads carrying crosslinked or adsorbed HisCPC were characterized in terms of size and Z-potential. Both crosslinking and adsorption of HisCPC to the beads resulted in an increased size of the beads (Figure 4A, samples 2 and 3, respectively). When compared to beads with adsorbed HisCPC, beads incubated with HisCPC and tyrosinase showed a wider size distribution (Figure 4B) and a lower Z-potential. A similar effect was seen on the HisCPC left in solution after the immobilization procedure. Soluble HisCPC incubated with beads and tyrosinase was crosslinked (Figure 4C inset, sample 2) had a slightly lower Z-potential than untreated HisCPC (sample 1) and HisCPC incubated with the beads but without tyrosinase (sample 3). We explained the decrease in Z-potential in the crosslinked beads and proteins as due to the accumulation of HisCPC that is negatively charged at pH 6.8, e.g. HisCPC has a calculated isoelectric point of 5.85, and the possible reduction in positively charged groups likely due to the reaction of uncharged tyrosines with positively charged amino groups, for example of lysines (pk_a_ = 10) or cysteines (pk_a_ = 8.3) during crosslinking with tyrosinase at pH 6.8.

## Discussion

The use of crosslinking enzymes such as tyrosinase, transglutaminase and peroxidase constitute an alternative to chemical crosslinkers in the production of protein aggregates. Enzymes have the advantage that they are used under mild conditions, have high specificity, and are non-toxic^1^. In addition, crosslinking occurs at specific site(s) of the protein and therefore is directed. However, limited availability and exposure of the target residues recognised by the crosslinking enzyme limit their application. Similarly to transglutaminase and peroxidase, studies on tyrosinases show that they are able to crosslink proteins with a loose structure or a not well-defined folding as these likely have accessible tyrosine side chains^22^,^24^,^25^,^26^,^27^. After being recombinantly produced and biochemically characterised, the well-folded fluorescent protein HisCPC was immobilized by crosslinking on solid surfaces through a tyrosine-containing, N-terminal peptide using the enzyme tyrosinase.

**Figure 5:**
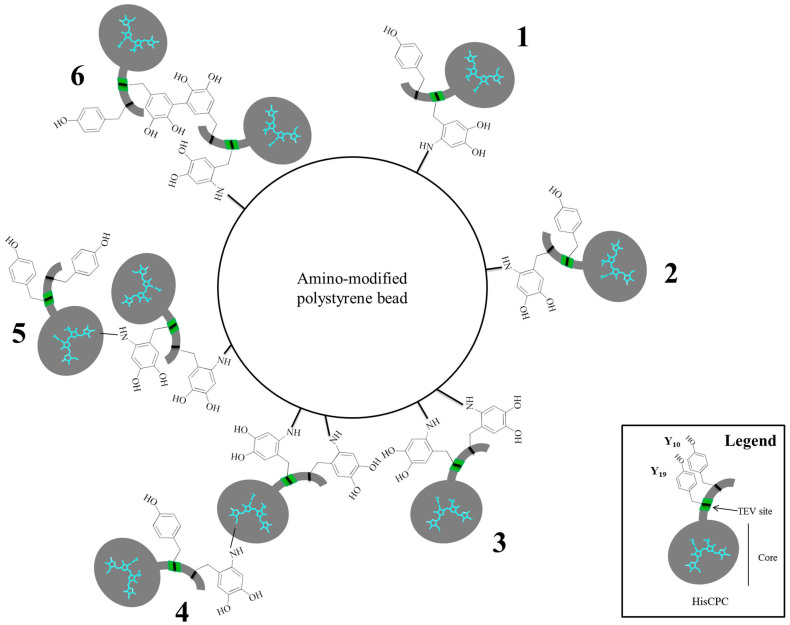
Schematic representation of the possible tyrosinase-catalyzed immobilization of HisCPC to amino-modified polystyrene beads according to our study. Crosslinking of HisCPC to the bead can involve Tyr10 **1** or Tyr19 that is located within the TEV cleavage site **2**, or both **3**. A second layer of HisCPC might be immobilized by introducing crosslinks between the nucleophilic residues from the HisCPC core and the N-terminal peptide of a second HisCPC molecule **4**, or free tyrosine residues from the N-terminal peptide of a bound HisCPC molecule and either nucleophilic groups from the HisCPC core **5**, or tyrosine residues of the N-terminal peptide **6**.

Crosslinking of HisCPC with tyrosinase in the presence of L-tyrosine resulted in a loss of fluorescence of the protein. This might be due to a quenching effect of melanin that is produced from tyrosine, as tyrosinase is the key enzyme of melanin biosynthesis. Also, it is possible that protein unfolding occurred caused by extensive inter-molecular crosslinking. However, such unfolding was not observed when CALB was similarly crosslinked in the presence of phenol^28^, e.g. a 30 mg/ml phenol concentration was added in order to crosslink CALB (2 mg/ml) with tyrosinase, as lipase activity was retained. However, that study pointed out how high phenol concentrations, e.g. 40 mg/ml, negatively affected the activity of the immobilized CALB^28^. In our study we used a lower ratio of phenolic compound to substrate protein, e.g. w/w ratio of 0.2 (0.5 mg/ml HisCPC and 0.1 mg/ml L-tyrosine) *versus* 15 (2 mg/ml CALB and 30 mg/ml phenol). However, direct crosslinking of HisCPC with tyrosinase in the absence of phenolic compounds led to dimer formation and an array of high molecular weight, covalently crosslinked protein aggregates. The HisCPC molecule carries many tyrosine residues that may function as primary and secondary sites of crosslinking by tyrosinase. Formed by an unstructured N-terminal peptide containing two tyrosines and a folded core with 10 tyrosines, the former was shown to be necessary for crosslinking of the protein with tyrosinase and its immobilization to amino-modified polystyrene beads. In fact, HisCPC carries various residues that can be secondary targets of crosslinking by the oxidised tyrosine, e.g. eight histidines, seven lysines, and one free cysteine. The production of an array of crosslinked HisCPC products rather than a single species, as shown in SEC analyses and SDS PAGE, is probably due to the complexity of the process. As for protein to protein crosslinking, immobilization to amino-modified polystyrene beads might also involve one (Figure 5 1 and 2) or both (Figure 5 3) the tyrosine residues of the N-terminal peptide. We cannot exclude that tyrosine residues not directly interacting with the surface react with other nucleophilic groups from a second HisCPC molecule (Figure 5 4, 5, 6) increasing the amount of proteins immobilized. Measurement of the size and Z-potential of the crosslinked beads showed the likely formation of multiple layers of HisCPC conferring a lower Z-potential and altered surface properties. Establishing precisely which of the two tyrosine residues from the N-terminal peptide is involved in the formation of the new covalent bonds is extremely challenging, as shown by studies performed using NMR and mass spectrometry with other proteins^22^ and beyond the scope of this study. However, future mutagenesis studies of HisCPC will be carried out. Hellman et al. also suggested how at neutral pH, e.g. at pH 6.8 as in our study, the formation of tyrosine-histidine links might be favoured over tyrosine-tyrosine ones, due to a change in nucleophilicity of the secondary amine of the histidinyl side chain^22^. However, unlike in this study, the spectroscopic characteristics after the reaction did not visibly change, or it was covered by the original strong blue colour of HisCPC.

This work shows that the production of enzyme-catalyzed covalently crosslinked, fluorescent, high-molecular weight aggregates of a fluorescent protein with possible application in the biomedical and diagnostic field is possible using a crosslinking enzyme such as tyrosinase instead of chemical crosslinkers. In line with previous studies showing the use of tyrosine-containing tags for enzymatic protein crosslinking^27^,^29^,^30^,^31^,^32^, we proved that tyrosines added to a protein within an unstructured region, e.g. a peptide, are not only specific sites of crosslinking but can also be used for site-specific protein immobilization. Tyrosine-containing tags can be easily engineered into a protein of choice and constitute interesting attachment sites for directed protein immobilization and surface functionalization.

## Methods

### Strains, materials, and enzymes

The bacterial strain used in this study is *E. coli* DH5α [genotype: F- *endA*1 *glnV44* *thi*-1 *recA*1 *relA*1 *gyrA*96 *deoR* *nupG* Φ80*acZ*ΔM15 Δ(lacZYA**-**argF)U169, *hsdR*17(rK^−^] mK+), λ^−^]. Unless otherwise stated, chemicals for buffer and gels preparation, commercial purified proteins and media components were purchased from Sigma-Aldrich (Switzerland). Tyrosinase from *Verrucomicrobium spinosum* (activity 1100 nkat/ml, specific activity 110 nkat/mg) was activated by trypsinization essentially as in^28^ but by adding 20 μg of TPCK-modified trypsin to 15 mg of pro-tyrosinase and it thus contained 1.5 fold less trypsin.

### Expression in shake-flask of HisCPC

Strain *E. coli* DH5α was transformed with both plasmids pAT101 and pBS414V and plated on selective agar plates containing spectinomycin (0.1 mg/ml) and kanamycin (50 μg/ml). For protein production, a pre-culture was set up overnight and used to inoculate a 300–500 ml culture that was induced with 1 mM IPTG at OD_600_ = 0.6. The culture media tested included LB medium (10 g/l tryptone, 5 g/l yeast extract, 10 g/l NaCl), TB medium (12 g/l peptone, 25.4 g/l yeast extract, 72 mM K_2_HPO_4_, 17 mM KH_2_PO_4_ pH 7.3, 4.0 g/l glycerol), and superbroth (32 g/l peptone, 2 g/l yeast extract, 5 g/l NaCl). A 2 l not-baffled flask containing 500 ml of selective medium was inoculated with a pre-culture to a final OD_600_ = 0.04 and incubated at 37°C, 180 rpm until an OD_600_ = 0.6 for LB medium cultures, or 1.2–1.3 for TB and SB medium cultures was reached and induction was performed with 1 mM IPTG, differently from^18^. Cells were harvested by centrifugation after 20 h at 4°C for 30 min at 4495 × g and the pellet stored at −20°C. Samples were withdrawn at regular intervals and, after pelleting the cells by centrifugation (5 min at 13000 rpm, 22°C), colour was evaluated and cells were used for SDS PAGE analyses. The influence of static incubation at different temperatures post-induction, and different OD_600_ at induction were assayed by inoculating a 2 l flask containing 500 ml of selective TB medium with a fresh pre-culture for an OD_600_ of 0.04. After incubation at 37°C, 180 rpm, protein production was induced at OD_600_ of 0.6 by addition of IPTG (1 mM final concentration). Cells were harvested by centrifugation after 20 h. Cultivations were performed in at least duplicates.

### Purification of HisCPC

HisCPC purification was performed as described in^33^. Briefly, cells were resuspended in 5 ml/g cell of 100 mM potassium phosphate pH 7.5, added of 1 mg/ml lysozyme and protease inhibitors EDTA-free (1 tablet/50 ml, Roche Complete Protease Inhibitor Mix, EDTA-free) and incubated on ice for 30 min before being frozen at −20°C. Once thawed, cells were treated with Benzonase Nuclease (Roche) for 30 min at 37°C under shaking conditions. Cells were placed on ice and subjected to 12 cycles of 10 s sonication with a Branson sonicator (Branson Ultrasonics, USA). Cell debris was removed by centrifugation for 40 min at 20000 rpm at 4°C with a Sorvall centrifuge (rotor SS34). HisCPC was purified by IMAC using an Äkta Purifier system and Histrap columns (GEHealthcare). Purification was performed in 100 mM potassium phosphate buffer at pH 7.5 and a step-wise increase in imidazole concentration, e.g. 50–100–200 mM imidazole. Blue fluorescent fractions were collected, concentrated and, buffer-exchanged to PBS by ultrafiltration (cut-off 10 kDa, Vivaspin, Sartorius) at 4000 rpm at 4°C in an Eppendorf 5810 centrifuge (Eppendorf, Switzerland).

### Spectrophotometric and fluorescence analyses

Spectrophotometric UV-Vis analyses were performed with a Cary Bio spectrophotometer equipped with a 96-well plate reader (Varian) using a 200 μl sample at 22°C. Fluorescence measurements were performed with a Cary Eclipse Fluorescence Spectrophotometer equipped with a multiwall-plate reader (Varian) in black 96-well plates with a 200 μl sample and with λ_ex_ = 609 nm and λ_em_ = 400–700 nm. Total protein concentration of cell extracts was estimated by measuring the absorbance at 280 nm, and CPC concentration was determined from the absorbance at 620 nm using an extinction coefficient ε^1%^_620_ = 70. As reference, the commercial C-phycocyanin from *Spirulina* sp. (*Arthrospira* sp.) was purchased from Sigma Aldrich (product nr. P2172, Switzerland).

### Biochemical characterisation of HisCPC and CPC

In order to assay the pH stability of HisCPC and CPC, protein solutions at a concentration of 0.05 mg/ml were prepared in McIlvaine buffer at pH 2.2–8 and in 100 mM Tris pH 9. Incubation was carried out for 5 min at 22°C. Temperature stability was determined by incubating protein solutions (0.05 mg/ml) in 100 mM potassium phosphate pH 7.5 in a temperature range of 22–80°C for 5 min. In both cases, protein integrity was assayed by measuring absorbance at 620 nm and the fluorescence at 640 nm, upon excitation at 609 nm. Size-exclusion chromatography was performed on a HiLoad 16/600 Superdex 200 prep grade column (GEHealthcare) in 100 mM potassium phosphate pH 7.5, 150 mM NaCl with a constant flow of 0.5 ml/min at 22°C. A 500 μl sample of 0.2 mg/ml protein solution was loaded. Elution of the proteins was monitored online at 280 nm and further analysed for absorbance at 620 nm with a Bio Tek Synergy Mx spectrophotometer. Molecular mass standards from the Gel Filtration Molecular Weight Markers Kit for Molecular Weights 12,000–200,000 Da (Sigma Aldrich) were used to calculate the calibration curve y = −1.8505x + 4.7947 with R^2^ = 0.90. CPC and HisCPC were quantified using an extinction coefficient at 620 nm of ε^1%^ = 70. SDS polyacrylamide gels were prepared with a 5 and 16% acrylamide content in the stacking and running gel, respectively. Proteins in the gel were stained with a Coomassie based solution (GelCode Blue Stain Reagent, Pierce) and imaged under white light. Bilin-binding proteins were visualised upon incubation for 5 min in 100 mM zinc acetate solution under UV light^34^. Imaging was performed with a GelDoc-It Gel Imaging Systems (UVP, Cambridge, UK).

### Crosslinking of HisCPC

Reaction mixtures contained 0.2–0.5 mg/ml HisCPC, 0.2 mg/ml tyrosinase and 0.1 mg/ml L-tyrosine, in 100 mM potassium phosphate buffer pH 6.8 and were incubated at 22°C under static conditions for either 70 or 100 min. Reactions were performed in triplicate. Changes in absorbance and fluorescence were measured as described above. Final reaction mixtures were stored at −20°C until analysed by SDS PAGE and size-exclusion chromatography using a 17 ml volume column packed with Superdex 75 at a constant flow of 1 ml/min in buffer 100 mM potassium phosphate pH 7.5, 150 mM NaCl at 22°C. The calibration curve y = −0.31x + 4.88 with R^2^ = 0.96, was obtained using proteins from the Gel Filtration Markers Kit for Molecular Weights 12,000–200,000 Da (Sigma Aldrich) as standards. In order to assess the thermal stability of crosslinked HisCPC, a 0.05 mg/ml HisCPC solution in 100 mM potassium phosphate pH 6.8 was incubated with 0.2 mg/ml tyrosinase at 22°C for 4 h under static conditions. Crosslinking was confirmed by assessing changes in the UV-Vis spectrum at ~310 nm. Native and crosslinked HisCPC were incubated at 30, 50, or 70°C for 15 min and the final sample was analysed for absorbance in a 260–700 nm range and for fluorescence (λ_ex_ = 609 nm and λ_em_ = 400–700 nm).

### immobilization of HisCPC

HisCPC at a 0.05 mg/ml concentration and pre-rinsed 16.8*10^8^ amino-modified polystyrene beads (Polybead® Amino Microspheres, 3.00 μm, 2.79%, Polyscience Inc.) were incubated in 100 mM K-phosphate pH 6.8 in the presence or absence of 20 μg of tyrosinase for four h at 22°C in a 50 μl final reaction volume. After rinsing three times with 400 μl 100 mM K-phosphate pH 6.8, beads were imaged with a Leica fluorescence microscope under bright light and with a N21 filter for fluorescence using a Leica Digital camera DFC350 FX (Leica, United Kingdom). Functionalized beads were then supplemented with either 1 ul TEV protease (Sigma Aldrich) at incubated at 30°C for four h or transferred to 400 μl 100 mM K-phosphate, 100 mM NaCl, 0.1% Tween20 pH 6.8 for 4 h at 22°C and imaged again. In order to assess the stability of HisCPC in 100 mM K-phosphate, 100 mM NaCl, 0.1% Tween20 pH 6.8, a 0.05 mg/ml protein solution was prepared in this buffer and incubated for 4 h at 22°C before recording a UV-Vis spectrum to assess protein integrity. Measurement of particle size and zeta potential (Z-potential) were carried out with a Zetasizer Nano ZS (Malvern, UK). Beads were prepared using a 0.5 mg/ml HisCPC concentration and 0.5 mg/ml tyrosinase. After being rinsed twice with buffer and eventually resuspended in 50 μl of 100 mM K-phosphate pH 6.8, the beads were added to 1 ml of deionized water and placed into a capillary cell to perform the measurement (final sample conductivity below 1.5 mS/cm). Each value is the average of at least three samples and the recordings were done at constant 25°C.

## Author Contributions

G.F. was responsible for planning, executing the experimental work, and drafting the manuscript. M.K. contributed to the discussion on HisCPC production. C.P. performed the experiments on HisCPC production and biochemical characterisation. All authors reviewed the manuscript and contributed to the discussion.

## Supplementary Material

Supplementary Information Supplementary Information
