# Supplementary material for: Tyrosinase-catalyzed site-specific immobilization of engineered C-phycocyanin to surface
Source: Sci Rep. 2014 Jun 20;4:5370. doi: 10.1038/srep05370 (PMC4064347; doi:10.1038/srep05370)
Supplement: Supplementary Information [file srep05370-s1.pdf]

Tyrosinase-catalyzed site-specific immobilization of engineered C-phycocyanin to surface

Greta Faccio[1]*, Michael M. Kämpf[1], Chiara Piatti[1,2], Linda Thöny-Meyer[1], Michael Richter[1]*

[1] Empa, Swiss Federal Laboratories for Materials Science and Technology - Laboratory for Bioactive Materials, Biocatalysis Group, Lerchenfeldstrasse 5, 9014 St. Gallen, Switzerland

[2] Dipartimento di Biotecnologie e Scienze della Vita, Università degli Studi dell'Insubria, via J.H. Dunant 3, 21100 Varese, Italy

***Corresponding authors**

Dr. Michael Richter
Group leader, Biocatalysis Group
Empa - Laboratory for Bioactive Materials
Lerchenfeldstrasse 5
CH-9014 St. Gallen (Switzerland)
Email: michael.richter@empa.ch
Tel. +41-587657792

Dr. Greta Faccio
Empa - Laboratory for Bioactive Materials
Lerchenfeldstrasse 5
CH-9014 St. Gallen
Switzerland
Email: greta.faccio@empa.ch
Tel. +41-587657262

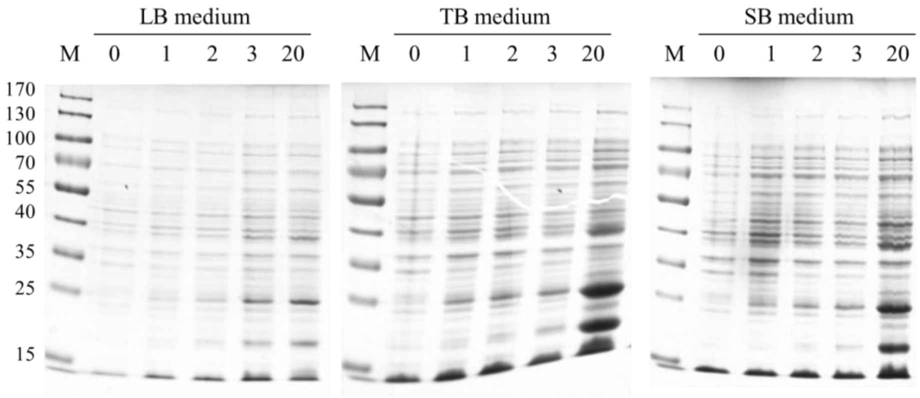

A

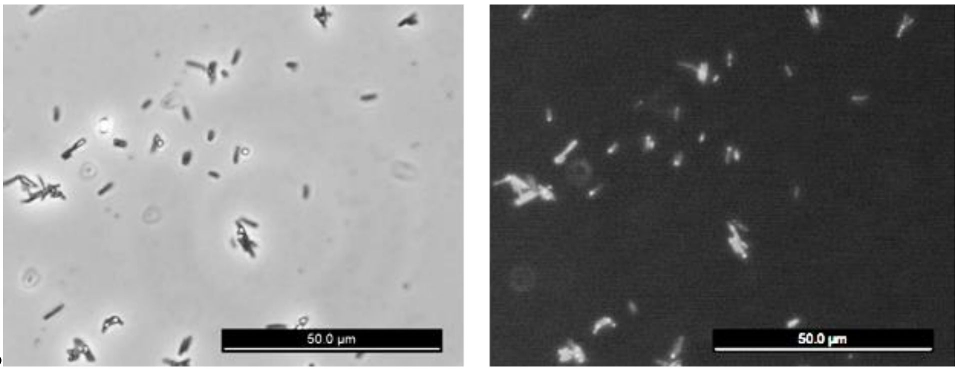

B

Supplementary Information S1 (A) SDS PAGE analysis of *E. coli* DH5α cells expressing HisCPC and cultured in LB, TB and SB media. 1 ml of culture was sampled at different intervals after induction (reported above each lane, in h). (B) *E. coli* DH5α cells producing recombinant HisCPC cultured in TB medium for 17 hrs. Cells visualised by phase contrast (left panel) and fluorescence with a N21 filter (λ_{ex}= 515-560 nm, λ_{em} = >590 nm, right panel).

Supplementary Information S2 Recombinant production of HisCPC in different conditions evaluated in the cell-free extract (CFE) and purified sample (values ± standard deviation).

Conditions	Temperature after induction (ºC)	Agitation (rpm)	HisCPC in CFE (mg/g cells)	Purified HisCPC (mg/g cells)	A_{620}/A_{280} of purified HisCPC	Purification Yield (%)
1	37	180	0.08±0.03	0.03±0.01	3.84±2.35	43%
2	22	0	0.37±0.10	0.16±0.04	1.31±0.22	45%
3	27	0	0.21±0.03	0.13±0.03	0.94±0.13	64%
4	32	0	0.18 ±0.04	0.08±0.02	1.14±0.12	45%
5	37	0	0.09 ±0.04	0.04±0.01	1.25±0.17	56%

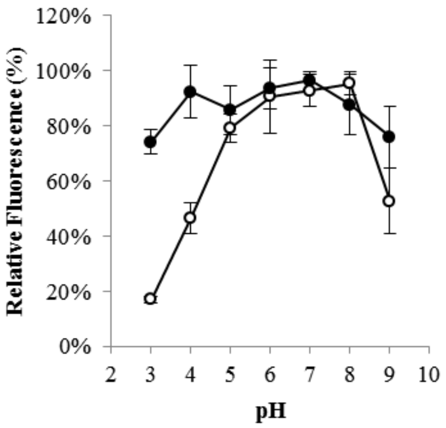

Supplementary Information S3 pH profile of recombinantly produced HisCPC (filled circles) and commercial CPC (empty circles) from *Spirulina* sp. as assayed by fluorescence.

Supplementary Information S4 Loss of fluorescence of HisCPC and CPC during crosslinking with tyrosinase in the presence of L-tyrosine. Reaction mixtures contained 0.2 mg/ml of protein, 0.1 mg/ml of L-tyrosine and 50 µg/ml of tyrosinase. Reactions were performed in triplicate at 22°C and monitored for 100 min (values ± standard deviation).

Sample	Residual fluorescence (%)	Fluorescence variation (ΔFluorescence/min)
CPC alone	90±1	-0.16±0.04
HisCPC alone	85±3	-0.77±0.26
Crosslinked CPC with L-tyrosine and tyrosinase	39±2	-0.86±0.06
Crosslinked HisCPC with L-tyrosine and tyrosinase	56±2	-2.33±0.04

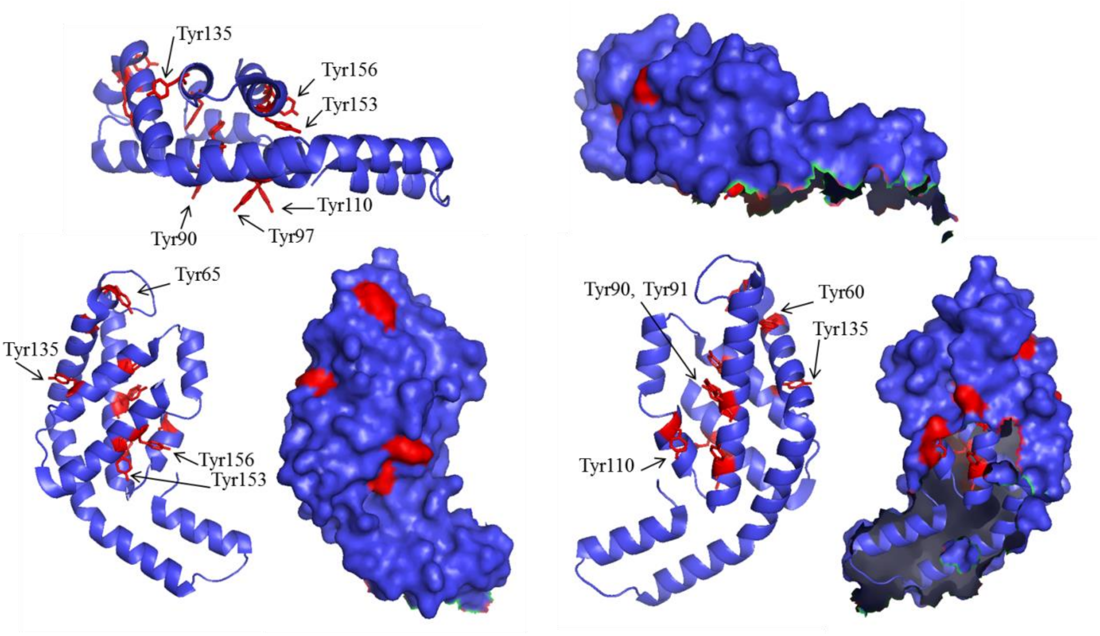

Supplementary Information S5 Three-dimensional structure of α-subunit of C-phycocyanin from *Synechocystis* sp. PCC 6803 (PDB ID: 4F0T) in a ribbon-stick and surface visualisation. The N-terminal 21 amino acid peptide present in HisCPC is not visualised. The molecule is shown from a side view (top) and from both sides (bottom). The backbone is in blue and tyrosine residues are reported in red as sticks. The incomplete surface of the molecule is due to the interaction with the beta subunit in the crystal. Images were prepared with Pymol (The PyMOL Molecular Graphics System, Version 1.5.0.4 Schrödinger, LLC.).
